# Gamma Knife^®^ icon CBCT offers improved localization workflow for frame‐based treatment

**DOI:** 10.1002/acm2.12745

**Published:** 2019-10-06

**Authors:** William N. Duggar, Bart Morris, Ali Fatemi, Jemeria Bonds, Rui He, Madhava Kanakamedala, Roberto Rey‐Dios, Srinivasan Vijayakumar, Claus Yang

**Affiliations:** ^1^ Radiation Oncology University of Mississippi Medical Center Jackson MS USA; ^2^ Radiology University of Mississippi Medical Center Jackson MS USA; ^3^ Neurosurgery University of Mississippi Medical Center Jackson MS USA

**Keywords:** CBCT, frame‐based, Gamma Knife, icon, radiosurgery workflow

## Abstract

**Object:**

The purpose of this study was to compare two methods of stereotactic localization in Gamma Knife treatment planning: cone beam computed tomography (CBCT) or fiducial. While the fiducial method is the traditional method of localization, CBCT is now available for use with the Gamma Knife Icon. This study seeks to determine whether a difference exists between the two methods and then whether one is better than the other regarding accuracy and workflow optimization.

**Methods:**

Cone beam computed tomography was used to define stereotactic space around the Elekta Film Pinprick phantom and then treated with film in place. The same phantom was offset known amounts from center and then imaged with CBCT and registered with the reference CBCT image to determine if measured offsets matched those known. Ten frameless and 10 frame‐based magnetic resonance imaging (MRI) to CBCT patient fusions were retrospectively evaluated using the TG‐132 TRE method. The stereotactic coordinates defined by CBCT and traditional fiducials were compared on the Elekta 8 cm Ball phantom, an anthropomorphic phantom, and actual patient data. Offsets were introduced to the anthropomorphic phantom in the stereotactic frame and CBCT's ability to detect those offsets was determined.

**Results:**

Cone beam computed tomography defines stereotactic space well within the established limits of the mechanical alignment system. The CBCT to CBCT registration can detect offsets accurately to within 0.1 mm and 0.5°. In all cases, some disagreement existed between fiducial localization and that of CBCT which in some cases was small, but also was as high as 0.43 mm in the phantom domain and as much as 1.54 mm in actual patients.

**Conclusion:**

Cone beam computed tomography demonstrates consistent accuracy in defining stereotactic space. Since both localization methods do not agree with each other consistently, the more reliable method must be identified. Cone beam computed tomography can accurately determine offsets occurring within stereotactic space that would be nondiscernible utilizing the fiducial method and seems to be more reliable. Using CBCT localization offers the opportunity to streamline workflow both from a patient and clinic perspective and also shows patient position immediately prior to treatment.

## 1 INTRODUCTION

With the advent of the Gamma Knife® (GK) Icon (Elekta, Sweden), image‐guided radiosurgery is now possible for this treatment unit. The Icon has the same delivery and patient positioning system as the Perfexion model, but has the addition of a cone beam computed tomography (CBCT) imaging arm and an intrafraction motion management (IFMM) system. The IFMM system is currently only applicable to patients immobilized with a thermoplastic mask rather than a frame, but the CBCT can be used for localization, or stereotactic space definition, for all patients, regardless of immobilization method. With the Icon and frame‐based treatments, stereotactic space may be defined using the traditional indicator boxes and imaging (CT, MR, or Angiography) or it can be defined by taking a reference CBCT of the patient with frame attached and in treatment position prior to plan approval. When stereotactic space is defined based on the localizer or indicator boxes, a pretreatment CBCT can be acquired in either a higher signal (CTDI 6.3) preset or a lower dose (CTDI 2.5) and registered with the stereotactically defined image set for comparison between patient coordinates at time of treatment to those at time of imaging. When space is defined with CBCT, the other imaging sets are co‐registered with the reference CBCT for coordinate definition and then another CBCT can be acquired directly before treatment to ensure stability of coordinate system. Initial evaluations of the GK Icon and its CBCT imaging have been quite encouraging regarding the continuation of gold standard accuracy.^1^, ^2^ The decision of clinical workflow with these new capabilities with the Icon depends on the comparison of the two methods of space definition regarding accuracy and efficiency. This study proposes that localization using CBCT may be more efficient, more effective at final treatment position validation than traditional localization using the indicator boxes during imaging, and at least comparable in accuracy.

## 2 SOURCES OF UNCERTAINTY

Traditional frame‐based GK utilizing the indicator box localization has been used successfully for decades in the treatment of thousands of cranial targets.^3^ Generally, system accuracy literature for this workflow has reported very lower error between 0.5 and 1 mm though with possible exceptions of up to 1.6 mm in special circumstances.^4^, ^5^, ^6^, ^7^, ^8^, ^9^ The confidence in this technique is high, but also the error margin is small as a “tolerable” accuracy limit has been proposed of 1.3 mm with emphasis that smaller is still better.^10^ The composition of the sources of this uncertainty looks different depending on which localization method is used and should be discussed.

Geometric distortion in MRI images must be discussed as the effect systematic to GK treatment planning regardless of localization method, but perhaps differs in magnitude of severity to which the accuracy is affected. Indeed, the MRI study has been identified as the most sensitive technical factor leading to the overall accuracy of the GK procedure, accounting for as much as 1 mm depending on the scan parameters and circumstances.^5^ Even with modern imaging capabilities, distortion in a phantom with the G‐frame attached is on average 0.53 mm with increasing magnitude with distance from the center of Leksell space (1.5 mm at 7 cm). This study by Pappas et al. indicates the frame’s presence itself as a major contributor of this distortion and also that severe distortions can occur outside the treatment area and affect accuracy with the fiducials in this space especially the lower portion near the frame.^11^ The further calculated dosimetric problems caused by this distortion are nontrivial and should be considered in addition to the discussion of the localization methods here.^12^ It should be noted that in general the intracranial distortion is smaller than voxels outside the cranium.

In evaluating the two methods, paramount importance exists in the understanding of the differences between them. Localization using the fiducials created by the indicator box sets the coordinate system at the time of imaging and then relies on the stability of the frame from imaging to treatment. As it turns out, this assumption is submillimeter from a systematic point of view, but could be a concern for random sources of error of more than 1 mm in some patients.^13^, ^14^, ^15^ Perhaps the greatest systematic sources of uncertainty for fiducial localization are that of imaging spatial resolution and fiducial identification accuracy and stability.^7^ Recently, significant frame distortion due to torque differential among fixation screws was demonstrated among even an experienced radiosurgery group which translated to inaccuracy in localization using the traditional indicator box method.^16^ In contrast, regarding localization with CBCT, the major sources of uncertainty will be the accuracy of the reconstructed stereotactic definition and the reliability of the image co‐registration between the CBCT images and those used for treatment planning, either CT or MR.

Image co‐registration has been addressed to a degree between MRI and CT within GammaPlan (Elekta, Sweden) in prior studies. Anatomic registration of multimodality images even in its early forms demonstrated statistically significant improvement in accuracy when co‐registering MRI to CT with fiducials, though perhaps the difference in slice thickness between the image modalities (3 mm for MRI to 1.5 mm for CT) may have been a major contributor.^17^ The current algorithm used in GammaPlan for co‐registration, Maximization of Mutual Information, demonstrates subvoxel accuracy between CT and MRI, with respect to the largest voxel size between the images. Interestingly, these same authors concluded that reduction of the previously discussed geometric distortion in MRI results in better co‐registration accuracy, indicating that the distortion indeed appears to be systematic through the co‐registration process as well.^18^ Watanabe and Han demonstrated the accuracy of co‐registration utilizing the current GammaPlan algorithm within the voxel size of the scanned images for the same modality and the registration accuracy for multiple modalities depends on the amount of common data between images, but was on the order of 1 mm, three‐dimensionally (3D).^19^ In the realm of modern imaging scanners and techniques, a final study found that the maximum co‐registration error between diagnostic MRI (no frame) and CT was <1 mm in any one direction while the max error was larger for stereotactic MRI (frame) co‐registered to CT, but still <1.6 mm. The authors concluded that registration accuracy appears to be limited by voxel size and MRI distortion.^20^

Admittedly, the aforementioned works have focused on CT and MRI registration instead of CBCT and in an earlier software version (10.1). Elekta has published a white paper on CBCT‐MRI demonstrating mean Target Registration Error (TRE) of less than or equal to 0.35 mm for CBCT co‐registration to 1 mm slice thickness MRI images for four separate patients and used synthetic CBCT and not an image acquired with the actual icon.^21^ Perhaps of more interest is the work of Chung et al. who compared the results of stereotactic definition through the indicator box and the CBCT co‐registration with MRI and found a statistically significant improvement of 0.4 ± 2 mm in utilizing CBCT‐MRI co‐registration over the indicator box.^22^ This work seeks to corroborate the accuracy and precision of the CBCT localization of the Icon, compare it to the alternative indicator box method, and then evaluate its ability to detect small changes in patient position within the stereotactic frame.

## 3 METHODS

This work involves patient data in some tests and is IRB‐approved, 2017‐0266.

### 3.1 Stereotactic definition of radiological focus using CBCT

**Figure 1:**
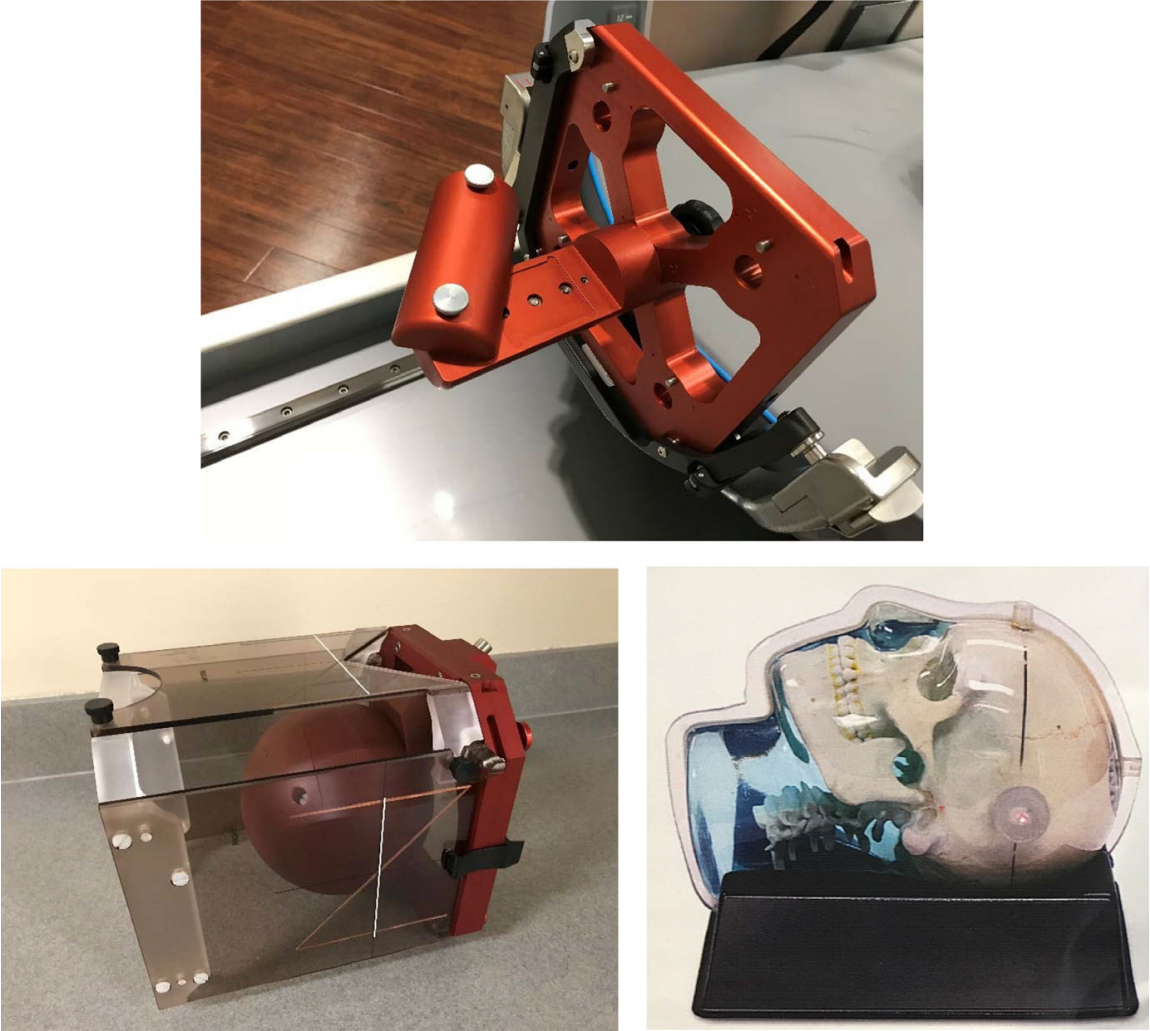
Phantoms utilized in this study. Top — Elekta Film Phantom Bottom Left — Elekta 8 cm radius Solid Water Phantom Bottom Right — Anthropomorphic Head Phantom.

The pinprick film phantom (Fig. 1) provided by Elekta underwent CBCT imaging (CTDI 6.3 — higher) as a stand‐alone CBCT in order to define stereotactic coordinates. These images were imported by GammaPlan (Version 11) and used as stereotactic definition of a treatment plan. Two shots were placed at around the visualized needle (around 100, 100, 100) to deliver dose for 1.8 min each. The plan was then exported to the GK delivery console. A film was placed in the phantom and pinpricked to define mechanical isocenter and the first shot was delivered. Between shots, delivery was paused and the phantom was rotated 90° and a new film was inserted and pinpricked before delivery of the second shot. This was repeated for a total of three measurements.

### 3.2 Stability and accuracy of stereotactic definition using CBCT

Once agreement between CBCT stereotactic definition and radiological focus was established, the same phantom was used to assess the definition across the range of potential stereotactic space. This phantom involves a cylindrical central insert that can be offset in both the X and Y directions by a distance of 6 cm. Therefore, the five positions are: (100, 100, 100), (160, 160, 100), (160, 40, 100), (40, 160, 100), and (40, 40, 100). Additionally, the phantom insert can be rotated 90° to around its axis. A reference CBCT was acquired with phantom at its central position. The phantom was then undocked and re‐docked into the G frame holder and three additional CBCTs at the central position were acquired and registered to the reference to assess alignment within treatment mode of GammaPlan. This was repeated with the phantom rotated 90° at center and then for each offset position. The measured discrepancies through registration were compared to the known offset values. The reference CBCT was acquired with CTDI setting 6.3 while follow‐up scans were performed with CTDI 2.5.

### 3.3 Co‐registration accuracy — MRI to CBCT

Ten frame‐based (MRI images with frame on) and 10 frameless (MRI images with no frame) patients were selected for image co‐registration analysis using AAPM TG‐132 methodology of target registration error (TRE) on the reference CBCT's and MRI planning images. Two separate investigators evaluated each image set by performing TRE for three different points in the fused datasets after co‐registration. The MRI images were T1 mprage with voxel size 1 × 1 × 1 mm. The MRI scanner utilized for this study was the Siemens MAGNETOM Aera 1.5 T Radiotherapy (Siemens Healthcare, Germany). Patient reference CBCTs were acquired with CTDI 6.3.

### 3.4 CBCT and fiducial localization comparison via registration

#### 3.4.1 8 cm Solid water ball phantom

The 8 cm radius Ball phantom (Fig. 1) with chamber and film inserts provided by Elekta was imaged with the fiducial box at CT simulation with normal clinical parameters (300 cm field of view and 1 mm slice thickness). The phantom was then treated as a patient and the images were imported into GammaPlan where the localization was defined based on the fiducials as for all frame patients. The phantom was placed in the G frame holder and set up as if treatment was to be delivered and then three CBCTs per CTDI setting were taken of the phantom and each was registered with the CT on which localization based on fiducials was defined. Registration was performed twice for each image set, once without a region of interest and then again with one. The discrepancies between CBCT localization and fiducial localization were recorded. The utilized CT Simulator was the Philips Brilliance Big‐Bore 16 Slice CT Scanner.

#### 3.4.2 Anthropomorphic phantom

The CIRS 3D Anthropomorphic Skull Phantom (Model 603A, Virginia, USA), shown in Fig. 1, was fixated with a stereotactic G frame and then underwent a reference CBCT and then MRI and CT imaging with the indicator box attached to the frame. The phantom images were then imported into GammaPlan and localization was defined on both the MRI and CT images based on fiducials. Then the phantom was docked into the GK Icon for CBCT imaging and three CBCTs were taken of the phantom and each was registered using a region of interest with all three of the initial image sets and the discrepancy recorded. The CBCT to CBCT registration at this point ensured that the phantom had not shifted within the frame. Then, known shifts of about 0.5 mm were applied by adjusting the screws of the head frame while the phantom was setup relative to lasers in the CT simulator room. The phantom was then returned to the Icon for docking and three more CBCT images. Cone beam computed tomography to CBCT registration at this point determined the expected shifts of the phantom after adjustment. The new CBCT images were also re‐registered with the original MRI and CT localization images. The new registration values were subtracted from the preshift registration values to show CBCT’s ability to detect small changes in actual patient position within the frame. Reference CBCTs were acquired with CTDI 6.3 while registration CBCTs were acquired with CTDI 2.5.

#### 3.4.3 Patient data

For frame‐based treatments, the localization has consistently been defined as traditional based on the fiducials of the localizer box after MRI and CT imaging. Prior to treatment, as both QA and data collection, a CBCT was taken of each patient and registered to one of the localization studies and the shifts were recorded. Descriptive statistics were performed on this data to determine consistency and look for trends. QA CBCTs were acquired with CTDI 6.3.

## 4 RESULTS

### 4.1 Stereotactic definition of radiological focus using CBCT

The accuracy of stereotactic definition of the radiological focal point (100, 100, 100) was determined to meet equivalent tolerance limits as applied to the acceptance testing of the patient positioning system (PPS) by itself of <0.4 mm. This is in agreement with another white paper published by Elekta reporting the accuracy to be a mean uncertainty of <0.2 mm with a weight of 70 kg on the couch.^23^ The results can be seen in Table 1.

**Table 1:** Agreement between cone beam computed tomography defined radiological focus and film test (deviation vector is the average of the three‐dimensional vector of each independent measurement).

Coordinate	∆X	∆Y	∆Z
100, 100, 100	−0.07 ± 0.03 mm	0.02 ± 0.17 mm	−0.1 ± 0.17 mm
Deviation vector	0.29 ± 0.06 mm		

### 4.2 Stability and accuracy of stereotactic definition using CBCT

The maximum discrepancy between the expected and measured distances was <0.15 mm and <0.5° demonstrating excellent stability and accuracy over a large area (12 cm × 12 cm in X and Y) of potential stereotactic space and again corroborating the results from Elekta.^23^ The full results can be seen in Table 2.

**Table 2:** Difference from expected phantom positions based on co‐registration with reference cone beam computed tomography of film pinprick phantom at stereotactic center (100, 100, 100).

	Rotation (°)			Translation (mm)		
	X	Y	Z	X	Y	Z
Position 100, 100						
Registration 1	−0.02	0.01	0.01	−0.02	0.01	0.01
Registration 2	0.00	0.01	−0.02	−0.02	0.00	0.02
Registration 3	0.00	0.00	−0.04	−0.02	0.00	0.01
Average	−0.01	0.01	−0.02	−0.02	0.00	0.01
Position 100, 100 R90						
Registration 1	−0.01	−0.08	−0.46	0.00	−0.08	0.02
Registration 2	−0.05	−0.06	0.21	0.01	−0.09	0.02
Registration 3	−0.05	0.00	−0.38	0.04	−0.07	0.05
Average	−0.04	−0.05	−0.21	0.02	−0.08	0.03
Position 160, 160						
Registration 1	−0.03	0.05	0.27	0.11	0.14	−0.02
Registration 2	−0.02	0.03	−0.09	0.09	0.14	0.04
Registration 3	0.00	0.02	0.25	0.09	0.14	0.04
Average	−0.02	0.03	0.14	0.10	0.14	0.02
Position 160, 40						
Registration 1	0.00	0.02	−0.03	0.09	−0.10	0.00
Registration 2	−0.04	−0.04	0.15	0.14	−0.10	0.03
Registration 3	−0.02	0.02	0.16	0.08	−0.07	0.01
Average	−0.02	0.00	0.09	0.10	−0.09	0.01
Position 40, 40						
Registration 1	−0.02	−0.04	−0.24	−0.06	−0.04	0.00
Registration 2	0.00	−0.02	0.12	−0.06	−0.04	−0.01
Registration 3	−0.02	−0.01	0.23	−0.06	−0.05	−0.05
Average	−0.01	−0.02	0.04	−0.06	−0.04	−0.02
Position 40, 160						
Registration 1	−0.03	−0.01	0.06	−0.10	0.05	−0.02
Registration 2	−0.02	0.00	0.22	−0.11	0.06	−0.03
Registration 3	−0.02	0.00	0.10	−0.10	0.05	−0.03
Average	−0.02	0.00	0.03	−0.10	0.05	−0.03

### 4.3 Co‐registration accuracy — MRI to CBCT

For observer 1 and frame‐based patients, the maximum 3D TRE was 1.17 mm and the 3D mean TRE was 0.7 ± 0.3 mm. For observer 2 and frameless patients, the maximum 3D TRE was 1.2 mm and the 3D mean TRE was 0.6 ± 0.2 mm. No significant difference existed between the two situations with *P* = 0.26. The full results can be seen in Table 3 compared with that of Chung et al.^22^

**Table 3:** Magnetic resonance imaging to cone beam computed tomography registration results (mm).

	Frame‐based	Frameless	Chung et al.
Deviation in X	0.4 ± 0.3	0.3 ± 0.3	0.1 ± 0.2
Deviation in Y	0.3 ± 0.3	0.3 ± 0.2	0.3 ± 0.3
Deviation in Z	0.3 ± 0.3	0.3 ± 0.2	0.3 ± 0.3
3D deviation	0.7 ± 0.3	0.6 ± 0.2	0.4 ± 0.2

### 4.4 CBCT and fiducial localization comparison

#### 4.4.1 Phantom studies

The various methods of comparison demonstrate some discrepancy between the localization methods, sometimes large (>0.5 mm) and sometimes small (<0.25 mm). For the 8 cm Ball phantom, some concern about material and shape homogeneity may exist which may affect registration consistency especially with rotations. Even so, a possible maximum discrepancy between fiducial localization and CBCT localization may exist for this phantom of somewhere between 0.7 and 0.94 mm in the Z direction. See Table 4 for the full results. Similar possible discrepancies can be seen with the anthropomorphic phantom.

**Table 4:** Difference between fiducial localization and cone beam computed tomography (CBCT) localization on the Ball phantom.

CTDI 2.5							CTDI 6.3						
	Rotation (°)			Translation (mm)				Rotation (°)			Translation (mm)		
	X	Y	Z	X	Y	Z		X	Y	Z	X	Y	Z
CBCT 1	0.46	−0.12	0.41	−0.18	−0.34	−0.94	CBCT 1	0.51	−0.02	−0.08	−0.13	−0.19	−0.64
CBCT 2	0.64	0.11	−0.03	−0.13	−0.27	−0.7	CBCT 2	0.24	0.08	−0.26	−0.17	−0.26	−0.54
CBCT 3	0.13	−0.31	0.02	0.08	−0.04	−0.81	CBCT 3	0.51	0.15	0.07	−0.12	−0.28	−0.79
Average	0.41	−0.11	0.13	−0.08	−0.22	−0.82	Average	0.42	0.07	−0.09	−0.14	−0.24	−0.66

In the anthropomorphic phantom test, it is important to note that a new CBCT image co‐registered to the pre‐CT and MRI reference CBCT demonstrates good agreement of <0.06° of rotation and <0.04 mm translation in any one direction which indicates the phantom did not shift in the frame during imaging. For MRI, rotational discrepancy was low for all comparisons (<0.25°), but a disagreement existed at a potential maximum of 0.43 mm in the x direction. Comparison to fiducial localization on CT was better in that no translation was observed larger than 0.24 mm. The rotations compared to CT were also smaller and no bigger than 0.15° in any direction. See Table 5 for the full results.

**Table 5:** Difference between fiducial localization and cone beam computed tomography (CBCT) localization for anthropomorphic phantom.

	Rotation (°)			Translation (mm)		
	X	Y	Z	X	Y	Z
MRI Sim with CBCT						
CBCT 1	0.12	−0.17	0.16	0.61	−0.17	−0.31
CBCT 2	0.25	−0.29	0.16	0.71	−0.13	−0.3
CBCT 3	0.22	−0.3	0.13	0.7	−0.12	−0.38
Average	0.197	−0.253	0.150	0.673	−0.140	−0.330
CT Sim with CBCT						
CBCT 1	0.11	0.06	0.13	0.24	−0.44	−0.18
CBCT 2	0.07	0.01	0.1	0.27	−0.42	−0.2
CBCT 3	0.06	0.05	0.09	0.26	−0.35	−0.08
Average	0.080	0.040	0.107	0.257	−0.403	−0.153

Abbreviation: MRI, magnetic resonance imaging.

After the roughly 0.5 mm shift was induced into the phantom position within the treatment frame, a CBCT was reacquired and co‐registered again with MRI and CT and the values were compared with the original co‐registration results. These results indicated that even a change as small as 0.5 mm could be discerned by CBCT registration with either MRI or CT as the error of the measured difference between co‐registration time points was <0.1 mm for all translational directions and <0.1° rotationally. See Table 6.

**Table 6:** Difference between expected phantom movement and detected phantom movement.

	Expected shifts (CBCT postshift registered to reference CBCT)						Expected — measured difference					
	Rotation (°)			Translation (mm)			Rotation (°)			Translation (mm)		
	X	Y	Z	X	Y	Z	X	Y	Z	X	Y	Z
MRI sim with CBCT												
−0.01		0.05	−0.07	0.03	−0.56	0.14	0.09	0.15	0.07	−0.04	0.01	0.04
							0.02	−0.01	−0.02	0.02	0.03	0.00
							−0.01	−0.00	−0.02	−0.02	0.11	0.15
				Average Difference			0.03	0.04	0.01	−0.02	0.05	0.06
CT sim with CBCT												
−0.01		0.05	−0.07	0.03	−0.56	0.14	−0.06	0.06	−0.10	−0.08	−0.03	0.02
							0.06	−0.26	−0.12	0.05	0.00	0.05
							0.07	−0.09	−0.08	0.03	0.03	−0.05
				Average Difference			0.02	−0.10	−0.10	−0.00	0.00	0.01

Abbreviations: CT, computed tomography; CBCT, cone beam CT.

#### 4.4.2 Clinical data

**Figure 2:**
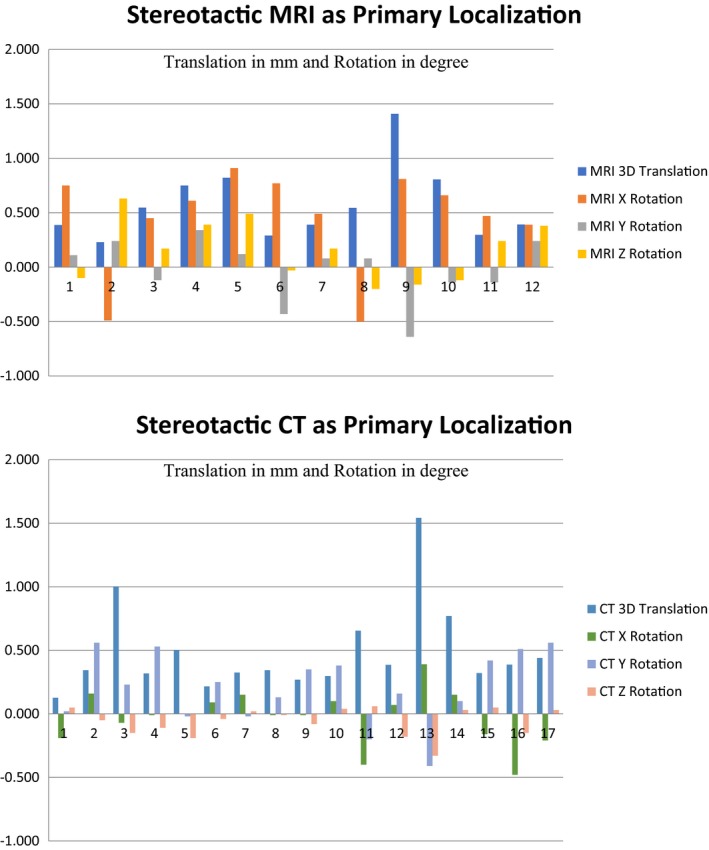
A comparison of fiducial localization to pretreatment cone beam computed tomography localization for patients separated based on primary dataset used for localization. Top — magnetic resonance imaging, Bottom — computed tomography.

Finally, the truth about the agreement between these two localization methods ultimately has the most impact for actual patients. Pretreatment CBCTs taken immediately prior to treatment and registered with either MRI or CT on 29 different patients of different target etiologies showed an average translational disagreement of 0.53 mm and a maximum for one case of 1.54 mm. Rotational agreement averaged 0.32°, 0.25°, and 0.16° around the X, Y, and Z axes, respectively. The maximum for any one patient was 0.91° around the X axis. Translation and rotation are calculated based on the origin or 100, 100, 100 and therefore shots furthest away from origin will be affected most by rotation whereas all shots will be affected equally by translation differences. Max shot displacement can be calculated and seen in the GammaPlan system and was collected for seven patients and ranged from 0.39 to 1.43 mm with an average of 0.67 mm. Figure 2 shows the discrepancies separated based on which image set the localization was defined. The differences were not statistically significant between MRI and CT regarding translation, but did show significance regarding average rotational differences though perhaps not practical significance. The full results can be seen in Table 7.

**Table 7:** A comparison of fiducial localization to pretreatment CBCT localization for actual patients with a mix of primary stereotactic reference image types (MRI and CT).

Patient site	X (mm)	Y (mm)	Z (mm)	X rotation (°)	Y rotation (°)	Z rotation (°)	3D translation (mm)	Primary stereotactic reference
1	−0.1	0.06	0.05	−0.19	0.02	0.05	0.13	CT
2	−0.23	−0.17	0.19	0.16	0.56	−0.05	0.34	CT
3	−0.85	0.25	0.47	−0.07	0.23	−0.15	1.00	CT
4	−0.01	0.22	0.23	−0.01	0.53	−0.11	0.32	CT
5	−0.15	0.35	−0.07	0.75	0.11	−0.1	0.39	MRI
6	0.05	0.2	−0.1	−0.49	0.24	0.63	0.23	MRI
7	0.45	−0.21	0.08	0	−0.02	−0.19	0.50	CT
8	0.15	−0.04	0.15	0.09	0.25	−0.04	0.22	CT
9	0.1	−0.39	−0.37	0.45	−0.12	0.17	0.55	MRI
10	0.27	0	0.7	0.61	0.34	0.39	0.75	MRI
11	0.57	−0.54	−0.24	0.91	0.12	0.49	0.82	MRI
12	0.06	−0.11	0.3	0.15	−0.02	0.02	0.33	CT
13	−0.19	−0.16	0.15	0.77	−0.43	−0.03	0.29	MRI
14	−0.28	−0.03	0.27	0.49	0.08	0.17	0.39	MRI
15	−0.04	−0.03	0.34	−0.01	0.13	−0.01	0.34	CT
16	−0.2	−0.06	−0.17	−0.01	0.35	−0.08	0.27	CT
17	0.14	0.17	0.2	0.1	0.38	0.04	0.30	CT
18	−0.5	0.08	−0.2	−0.5	0.08	−0.2	0.54	MRI
19	0.49	−0.11	−0.42	−0.4	−0.2	0.06	0.66	CT
20	−0.35	0.11	0.12	0.07	0.16	−0.18	0.39	CT
21	−1.21	0.24	0.68	0.81	−0.64	−0.16	1.41	MRI
22	−1.27	0.09	0.87	0.39	−0.41	−0.33	1.54	CT
23	0.41	−0.44	0.48	0.15	0.1	0.03	0.77	CT
24	0.17	0.22	0.16	−0.16	0.42	0.05	0.32	CT
25	−0.14	−0.75	−0.26	0.66	−0.14	−0.12	0.81	MRI
26	−0.28	−0.06	−0.08	0.47	−0.14	0.24	0.30	MRI
27	0.34	0.19	0.04	0.39	0.24	0.38	0.39	MRI
28	−0.08	0.36	−0.12	−0.48	0.51	−0.15	0.39	CT
29	0.29	0.16	0.29	−0.21	0.56	0.03	0.44	CT
CT average (absolute)	−0.06	0.03	0.19	−0.03	0.21	−0.06	0.49	
CT maximum (absolute)	0.49	0.36	0.87	0.39	0.56	0.06	1.54	
MRI average (absolute)	−0.12	−0.07	0.04	0.44	−0.02	0.16	0.57	
MRI maximum (absolute)	0.57	0.35	0.70	0.91	0.34	0.63	1.41	
Significance between MRI and CT (*P* − value)	0.72	0.32	0.23	0.00	0.04	0.01	0.51	
Total average (absolute)	0.11	0.01	0.16	0.32	0.25	0.16	0.52	
Total maximum (absolute)	1.27	0.54	0.87	0.91	0.56	0.63	1.54	

Abbreviations: CT, computed tomography; CBCT, cone beam CT; MRI, magnetic resonance imaging.

## 5 DISCUSSION

### 5.1 Risks of traditional localization and advantage of CBCT

Though the traditional methods of localization have garnered the confidence of multiple decades of clinical use, this study in addition to the work of others has demonstrated that frame distortion and/or slippage is possible, though perhaps rare.^15^ The reasons for large discrepancies for a few patients are not fully clear from this study. Perhaps, the frame was distorted from torque differential across fixation screws such as demonstrated by Renier and Massager.^16^ Actual frame slippage or mounting errors may also explain the discrepancies in our study as in the study of Peach et al.^15^ While the cause may be uncertain, the presence of these uncertainty risks remains while the use of CBCT localization ensures stereotactic definition while the patient is docked into treatment position which will prevent these random errors from causing targeting inaccuracy.

### 5.2 Validation of CBCT Localization and proposed workflow improvements

As an isolated system, CBCT imaging on the Icon demonstrates impressive stability and accuracy of localization of the treatment area, adding almost no additional uncertainty to that demonstrated by the positioning system itself. The weakness of the CBCT method is perhaps its reliance on image co‐registration with planning images (either CT or MR), but the uncertainty of this image co‐registration may be as low as 0.4 mm.^21^ Additionally, the co‐registration accuracy appears to be primarily limited by image voxel size and MRI distortion which are both systematic throughout the process with either localization method. Though our data did not completely agree with that of Chung et al., it was at least comparable to both their data for co‐registration and indicator box localized studies.^22^ There is perhaps still a modest improvement for CBCT to MRI compared to fiducially localized studies when comparing to their values, though not as pronounced nor necessarily significant.^5^, ^18^, ^20^ It should be noted that Chung et al. used MRI images with smaller voxel sizes than those used in our study.^22^ Therefore, it appears that workflows using CBCT for localization lead to clinically acceptable and comparable to fiducial‐based dosimetric accuracy.^10^

Since CBCT localization is at least comparable to fiducial‐based, the workflow for GK can be improved in several ways when needed. First, thresholds could be established based on known uncertainty of the CBCT co‐registration for which shift values above that threshold will be corrected by replanning and utilizing CBCT as the stereotactic space definition. This would lead to higher confidence in the delivery of the treatment than prior to the Icon, pretreatment imaging and verification was not possible, especially since the ability of CBCT on the Icon to detect small, but clinically significant changes in patient position has been demonstrated. The validation of the Icon CBCT as a pretreatment QA device extends beyond this work as well.^24^ Secondly, the localization using fiducials could be abandoned. Since CBCT localization appears to be comparable in accuracy, a reference CBCT can be acquired once the frame has been placed as a part of pretreatment imaging. The advantage of this approach is multifactorial in that now frame placement is only necessary prior to the CBCT scan. MRI and/or CT images could be acquired and a plan generated without the frame. This workflow drastically reduces the amount of time that the patient wears the frame and also increases flexibility with scheduling as the entire workflow is not required to occur over a single day. Also, CBCT localization may be more accurate due to lesser distortion without in MRI images without the frame included and ultimately less systematic uncertainty.^11^, ^20^ A pretreatment CBCT in this case represents only CBCT uncertainty (very small as seen above) and actual patient movement within the frame. The preplanning workflow with CBCT still represents an improvement over previous preplanning workflows as those still required a treatment day MRI or CT with the frame in place, while CBCT is faster to acquire and is done so with the patient in treatment position. Assuming the treatment plan is ready at this time, only a few processing steps remain after CBCT acquisition and treatment can be started within a short time (usually <10 min).

The use of CBCT in GK treatment has only just begun, but the possibilities and improvements of using actual anatomy to define stereotactic space are perhaps just now being understood. The CBCT unit on the Icon has demonstrated great stability and accuracy. Some challenges and opportunities for improvement still exist in image co‐registration, but the potential new workflow of using CBCT for localization has pertinent and important ramifications for not only frameless patients, but also those treated with the traditional frame. The GK community must seek out opportunities for improvement utilizing this new generation of GK treatment devices.

## 6 CONCLUSIONS

Based on the work and discussion above, it appears that CBCT on the GK Icon offers an alternative to the traditional localization workflow. Utilizing CBCT for stereotactic space definition does not sacrifice systematic accuracy of the frame‐based treatment in lieu of traditional localizer methods. In some cases, where higher than normal uncertainty may be experienced by some patients using traditional methods, CBCT localization improves upon traditional methods by defining stereotactic space at the time of patient docking into the treatment unit which is the most crucial time in the treatment workflow. Cone beam computed tomography localization improves upon traditional methods by offering a more patient‐centric workflow which requires less time in the treatment frame and less time in the clinic on treatment days. Cone beam computed tomography localization for frame‐based patients can replace traditional methods for clinics utilizing the GK Icon.

## CONFLICTS OF INTEREST

The authors have nothing to disclose.
